# A Case of Right Subclavian Artery Agenesis

**DOI:** 10.1055/s-0042-1743534

**Published:** 2022-11-01

**Authors:** Rose C. Gooding, George L. Hines

**Affiliations:** 1 Department of Surgery, New York University Langone Hospital – Long Island, Mineola, New York

**Keywords:** subclavian artery, agenesis, aortic arch, anomaly

## Abstract

The authors present a 12-year-old male with an asymptomatic absence of the proximal right subclavian artery. On physical examination, his right brachial, radial, and ulnar pulses were diminished compared with the left side. Computed tomographic angiography revealed that the right subclavian artery was supplied from the anterior right internal mammary artery. A description of the acquired and congenital aortic arch anomalies and thoracic outlet syndrome differential diagnoses is provided.

## Introduction

Unilateral absence of a brachial, radial, and ulnar pulse in a pediatric age patient is rare. Possible etiologies include congenital abnormalities, trauma, vasculitis, embolus, or possibly sequelae of arterial thoracic outlet syndrome. We present an asymptomatic 12-year-old patient who, on examination, was found to have no palpable right radial or brachial pulse.

## Case Presentation

A 12-year-old male presented to the emergency department for evaluation after a concussion, and there was reported difficulty in palpating his right radial artery. He was sent to a vascular surgeon for evaluation where blood pressure was 125/80 mm Hg in the left arm and 80/40 mm Hg in the right arm. On physical examination, his right brachial, radial, and ulnar pulses were decreased as compared with the left. The patient had no acute medical concerns and denied ischemic symptoms in the arm.

**Fig. 1:**
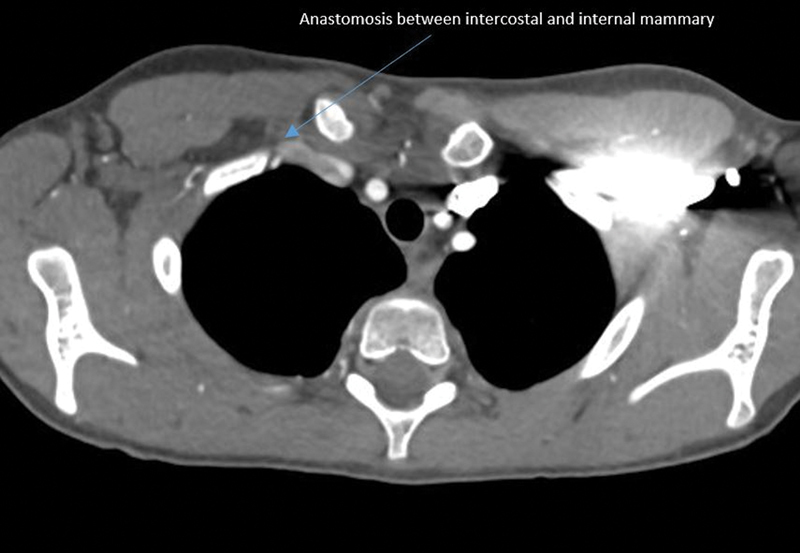
There is an anastomosis between the intercostal and internal mammary.

**Fig. 2:**
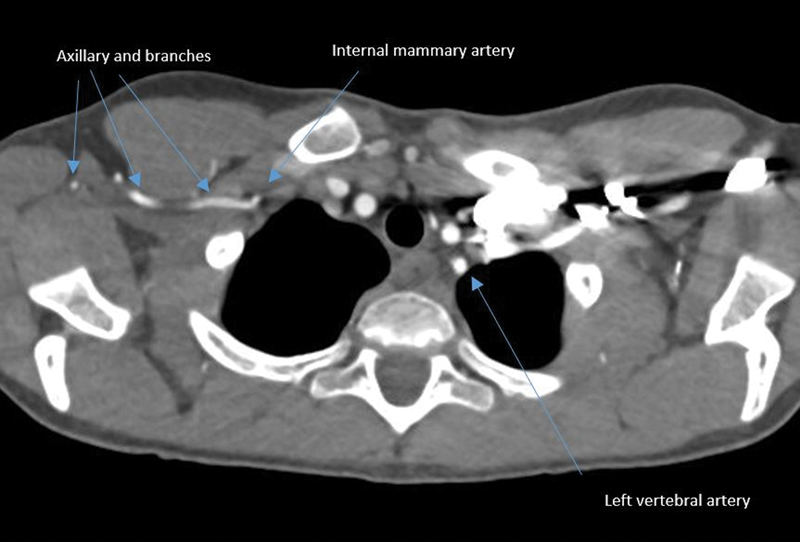
Right vertebral artery is not present.

**Fig. 3:**
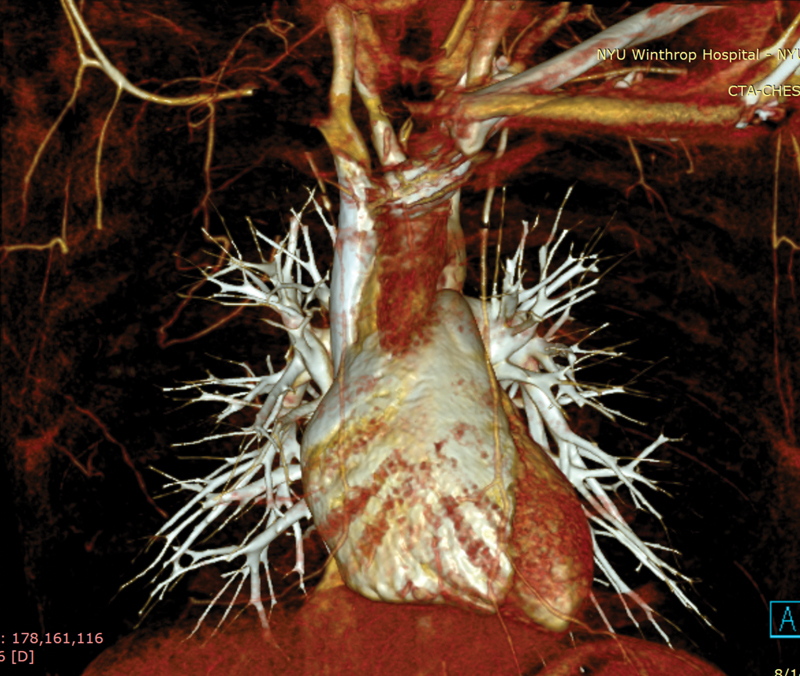
Three-dimensional reconstruction of aortic arch and thoracic vessels.

A chest X-ray demonstrated normal anatomy and no evidence of a cervical rib. Computed tomography angiography (CTA) of the chest revealed that the right subclavian artery did not have an origin from the brachiocephalic artery but rather filled distally from the right internal mammary artery ( Fig. 1 ). There was no definitive soft tissue mass at the thoracic outlet region and there were no cervical ribs. Interestingly, there was also no vertebral artery identified on the right side ( Fig. 2 ). A three-dimensional reconstruction was performed from the CTA showing a discrete area of absence of the right subclavian artery ( Fig. 3 ).

## Discussion

**Fig. 4:**
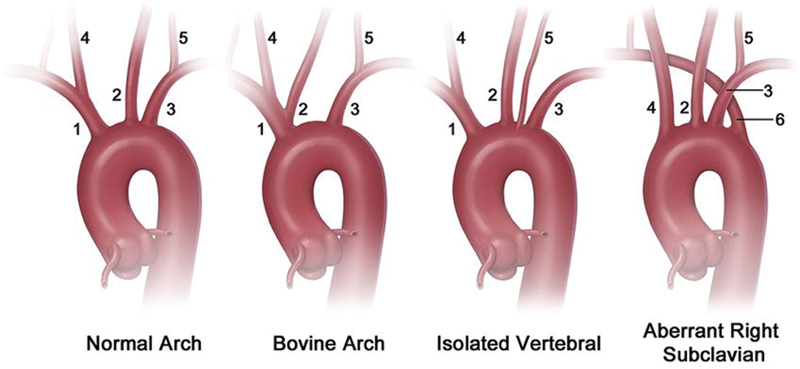
Aortic arch anomalies. 1, brachiocephalic artery; 2, left common carotid artery; 3, left subclavian artery; 4, right common carotid artery; 5, left vertebral artery; 6, aberrant right subclavian artery. Reproduced with permission from Dumfarth et al. 3

Aortic arch anomalies are present in 35% of the population and result when segments of the primitive aorta do not disappear and persist, or vice versa. 1 Most commonly, the left common carotid artery originates from the brachiocephalic trunk rather than from the aortic arch and is termed a bovine arch as in ruminant animals. In this most common arch anomaly, the brachiocephalic trunk gives rise to the right subclavian, right common carotid, and left common carotid arteries, leaving the left subclavian to originate directly from the aortic arch. The remaining variations occur in less than 3% of the population. 1 These are reported as follows: First, a shortened brachiocephalic trunk can bifurcate immediately into the right subclavian and right common carotid arteries with the left common carotid arising from the aortic arch at the base of the brachiocephalic trunk and a normal origin for the left subclavian artery from the aortic arch. Second, the left vertebral artery can originate directly from the aortic arch between the left common carotid and subclavian arteries. Third, the aortic arch can be right sided, and the right subclavian artery can be retroesophageal, and termed the arteria lusoria. Also, in this setting, the isolated left subclavian artery fills by retrograde flow from the left vertebral artery creating a congenital left subclavian steal. 2 Most of these anomalies are asymptomatic except for the aberrant right subclavian artery in which 5% of patients have compression of the esophagus causing difficulty in swallowing resulting in the condition, dysphagia lusoria. This anomaly is also prone to aneurysmal development, Kommerell diverticulum, or a bulbous configuration of the origin of an aberrant left subclavian artery which can lead to compressive symptoms, thromboembolism, or rupture. An image recreated to visualize these anomalies has been provided ( Fig. 4 ). 3

The innominate artery is formed by the right horn of the aortic sac and the left becomes the proximal ascending aorta. The 3rd aortic arch forms the common carotid arteries while the 4th aortic arch separates and forms a left and right portion. The left portion of the 4th aortic arch forms the area of the aortic arch between the left common carotid and the left subclavian arteries. The right portion of the 4th aortic arch becomes the proximal segment of the right subclavian artery with root segments three through seven. The distal aspect of the proximal right subclavian artery is derived from the right dorsal aorta and its right 7th intersegmental artery. Also arising from the right 7th is the right vertebral artery.

Subclavian arteries are the embryological product of 7th intersegmental arteries. 4 During normal embryogenesis, the distal segment of the right dorsal aorta involutes, allowing the 4th vascular arch and the 7th intersegmental artery on the right side to become the brachiocephalic and the subclavian arteries. 4 In 0.5% of population, the right 4th vascular arch and proximal right dorsal aorta involute, leaving the right 7th intersegmental artery attached to the left descending aorta via the distal part of the right dorsal aorta forming the aforementioned Kommerell's diverticulum.

Our other differential diagnoses consisted of thoracic outlet syndrome, an embolic event, vascular agenesis, some type of vasculitis, or trauma. The arterial form of thoracic outlet compression is present in less than 1% of cases of thoracic outlet syndrome. 1 Patients present with microembolization to the hand and ischemic symptoms. This disease is defined by the presence of arterial compression, poststenotic dilation, aneurysmal degeneration, and a secondary embolism. The compression can be secondary to a cervical or anomalous first rib, clavicular fracture, or anomalous insertion of the anterior scalene muscle. This syndrome is evaluated using duplex ultrasound looking for an aneurysm, thrombus, turbulence, and elevated velocities, or CTA with the patient's arms at the sides and again hyper abducted and externally rotated.

Few reports of vascular agenesis exist in current literature, with the most relevant to our case being unilateral congenital absence of the internal carotid artery (ICA). So far eight cases of bilateral total absence of the ICA, and seven cases of unilateral partial congenital absence of the ICA with an intercavernous anastomosis between the contralateral “normal” and ipsilateral partially aplastic ICA have been identified by angiography. 5 Events that lead to absence of the carotid arteries is unknown. These anomalies are usually identified during angiography for patients who present with intracerebral hemorrhage as they have concurrent intracerebral aneurysms. 5

We believe our patient may have had an intrauterine vascular insult to the 7th intersegmental arteries or to the 4th aortic arch thus causing there to be an absence of the right subclavian artery and right vertebral artery. What would be the subclavian or proximal axillary artery is formed by a branch of the internal mammary and an anterior continuation or a posterior intercostal artery. A few of the branches appear hypertrophied including the lateral thoracic artery and the anterior intercostal, likely as a response to demand from the arm. The patient has no symptoms, and therefore further investigation will not be undertaken.
